# Effects of Immune System-Related Medications on COVID-19 Outcome in a Cohort of Iranian Patients: Preliminary Report of a Data Mining Study

**DOI:** 10.1155/2021/9934134

**Published:** 2021-07-02

**Authors:** Ehsan Bitaraf, Seyyed Amir Yasin Ahmadi, Alireza Gandomi-Mohammadabadi, Zahra Noorani Mejareh, Bahare Abdollahi, Javad Balasi, Farzan Moodi, Nima Hemmati, Ali Kabir

**Affiliations:** ^1^Center for Statistics and Information Technology, Central Library, Iran University of Medical Sciences, Tehran, Iran; ^2^Student Research Committee, School of Medicine, Iran University of Medical Sciences, Tehran, Iran; ^3^Minimally Invasive Surgery Research Center, Iran University of Medical Sciences, Tehran, Iran

## Abstract

**Background:**

Regulation of the immune system is critical for fighting against viral infections. Both suppression and hyperactivity of the immune system result in failure of treatment. The present study was designed to show the effects of immune system-related medications on mortality and length of stay (LOS) in a cohort of Iranian patients with coronavirus disease 2019 (COVID-19).

**Methods:**

A data mining study was performed on 6417 cases of COVID-19 covered by 17 educational hospitals of Iran University of Medical Sciences, Tehran. Association of a researcher-designed drug list with death and LOS was studied. For death outcome, logistic regression was used reporting odds ratio (OR) with 95% confidence interval (CI). For LOS, right censored Poisson regression was used reporting incidence rate ratio (IRR) with 95% CI.

**Results:**

Among the corticosteroids, prednisolone was a risk factor on death (OR = 1.41, 95%CI = 1.03 − 1.94). This association was increased after adjustment of age interactions (OR = 3.45, 95%CI = 1.01 − 11.81) and was removed after adjustment of ICU admission interactions (OR = 2.64, 95%CI = 0.70 − 9.92). Hydroxychloroquine showed a protecting effect on death (OR = 0.735, 95%CI = 0.627 − 0.862); however, this association was removed after adjustment of age interactions (OR = 0.76, 95%CI = 0.41 − 1.40). Among the antivirals, oseltamivir showed a protecting effect on death (OR = 0.628, 95%CI = 0.451 − 0.873); however, this association was removed after adjustment of age interactions (OR = 0.45, 95%CI = 0.11 − 1.82). For reduction of LOS, the only significant association was for hydroxychloroquine (IRR = 0.85, 95%CI = 0.79 − 0.92).

**Conclusion:**

The results of such data mining studies can be used in clinics until completing the evidence. Hydroxychloroquine may reduce mortality in some specific groups; however, its association may be confounded by some latent variables and unknown interactions. Administration of corticosteroids should be based on the conditions of each case.

## 1. Introduction

Coronavirus disease 2019 (COVID-19) which is caused by severe acute respiratory syndrome coronavirus 2 (SARS-CoV-2) was first found in Wuhan, China, In December 2019. According to a report, about 87.9% of hospitalized patients had fever and severe pneumonia occurred in 15.7% of patients [1].

Clinically, there are two phases of the immunologic responses induced by SARS-CoV-2 infection: antiviral response and inflammation response. Potent immune response could eliminate the virus and prevent disease progression to severe stages in incubation period and at nonsevere stages. Therefore, at nonsevere stage, it is important to enhance immune responses. It seems that in immunocompromised patient, virus spread rapidly and leads to massive destruction in the affected organs [2]. Thus, the main cause of the delayed critical cascade of uncontrolled immune events may be the suppressed antiviral innate immune response induced by the virus and activation of the proinflammatory cytokines which lead to fulminant systemic inflammation [3]. The damaged cells induce innate inflammation by proinflammatory macrophages and granulocytes in the lungs. Lung inflammation is the main cause of respiratory disorders which is life-threatening at the severe stage [4].

When COVID-19 progresses from severe to critical, patients may develop a severe cytokine storm and secondary acute respiratory distress syndrome, followed by shock, tissue perfusion disorders, and even multiorgan failure [1]. Although cytokine storm limits further spread of virus in the body, it induces secondary tissue damage through the secretion of large amounts of active mediators and inflammatory factors. It is one of main causes of death in COVID-19 patients; hence, it has been considered as a critical therapeutic target. Although the exact mechanism of cytokine storm is not understood yet, overactivation of the innate immune system and imbalanced angiotensin converting enzyme 2 (ACE2) expression seem to play a key role. Several clinical trials were conducted with the purpose of controlling cytokine storm. However, some questions need to be answered before widespread use of these treatments, i.e., what are the adverse effects of these therapeutic methods? When is the best time to start them? Are they the best possible treatment?

Regretfully, severe COVID-19 still has unclear pathophysiology and treatment. There are some uncertain immunomodulatory or immunosuppressive treatments for severe types of COVID 19 such as hydroxychloroquine, interleukin (IL)-6, and IL-1 antagonists [4]. There is currently no specific treatment drug to target SARS-CoV-2. So, deciding on potential treatment regimens for the prevention and treatment of severely ill COVID-19 patients remains a major challenge [5]. Briefly, the most important challenge is when to suppress and when to enhance the immune system through the current common drugs.

According to the above questions and limitations, we studied a cohort of Iranian patients to evaluate the effect of immune suppressants, modulators, and stimulants as well as some antivirals and antibiotics on mortality and length of stay (LOS) in patients with COVID-19.

## 2. Material and Methods

### 2.1. Study Design

A data mining project was conducted in the datacenter of Iran University of Medical Sciences, Tehran, Iran. The data was obtained from the 17 educational hospitals covered by the university between 20 March 2020 and 11 August 2020.

The inclusion criteria were being confirmed case of COVID-19 by the government with ICD-10 code U07.1 and being hospitalized. The exclusion criteria for inferential analysis were discharge from hospital by personal consent, escape from hospital, or transfer to another center.

### 2.2. Variables and Definitions

Background variables: The governmental ID of the patients was used to merge the other variables as a uniform dataset. Age, gender, intensive care unit (ICU) admission, date of admission, and condition on discharge were other background variables. The categories of condition on discharge were complete remission, relative remission, death, escape from hospital, discharge by personal consent, and transfer to another center. Only the patients in the first three categories of condition on discharge were regarded for inferential statistical analysis.

The drugs of study: A researcher-designed drug list was prepared. The names of the variables were as the following. Sofosbuvir (indicating sofosbuvir/daclatasvir), lopinavir (indicating lopinavir/ritonavir), ribavirin, favipiravir, oseltamivir, interferon, metronidazole, linezolid, dexamethasone, hydrocortisone, budesonide (as nebulizer), prednisolone, methylprednisolone, infliximab, mycophenolate, IVIG (intravenous immunoglobulin), vitamin C, azathioprine, ciclosporin, tacrolimus, aspirin, warfarin, clopidogrel, acetaminophen, diphenhydramine, antivirals (indicating any kind of antiviral), CSF (colony stimulating factor), cephalosporins, macrolides, fluoroquinolones, lincosamides, glycopeptides, carbapenems, cotrimoxazole, glucocorticoids (any kind of glucocorticoid), heparin_group (indicating unfractionated heparin and enoxaparin), and hydroxychloroquine.

Outcome variables: The outcome variables were death and LOS. Death was a category of the variable condition on discharge, and LOS was the difference between admission date and discharge date.

Generated variables: Some new variables were designed and generated by the researchers. *Death* was generated as a binary variable from condition on discharge. *Age group* was generated from age. *Underlying drugs* was a binary variable indicating receiving of at least one of the drugs showing underlying infectious or cardiovascular disease of a patient or having diabetes (linezolid, warfarin, clopidogrel, fluoroquinolones, lincosamides, glycopeptides, carbapenems, cotrimoxazole, insulin, or oral antidiabetic agents).

### 2.3. Data Source

The source of the primary data was Iranian integrated care electronic health record (locally called SEPAS). SEPAS is a repository for storing health-related information for each individual. This repository has been created at the national level, in Iran.

### 2.4. Association Rule Mining

**Figure 1:**
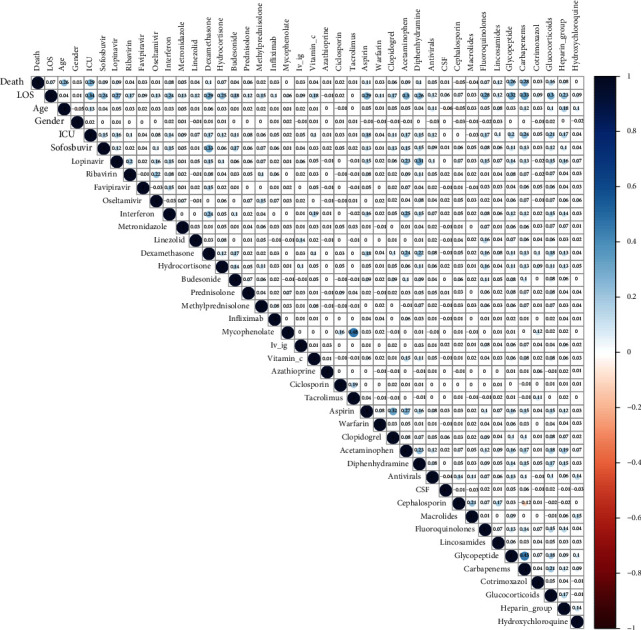
Correlation plot for association rule mining.

**Figure 2:**
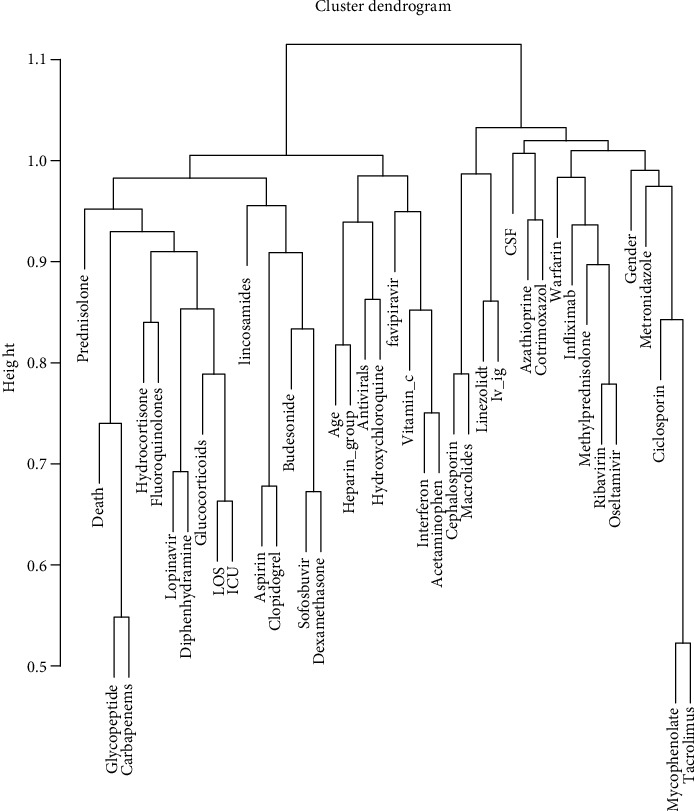
Dendrogram for association rule mining.

This process was performed to study all the possible two by two associations between the variables. Correlation plot and dendrogram were used to show accompaniment of the variables (Figures 1 and 2). Support and confidence methods were also used. Support (variable⟶death) was defined as the probability of simultaneous positivity of the variable and death in all the samples. Confidence (variable⟶death) was defined as the probability of simultaneous positivity of the variable and death in all the patients with positivity of that variable.

### 2.5. Data Clustering

**Figure 3:**
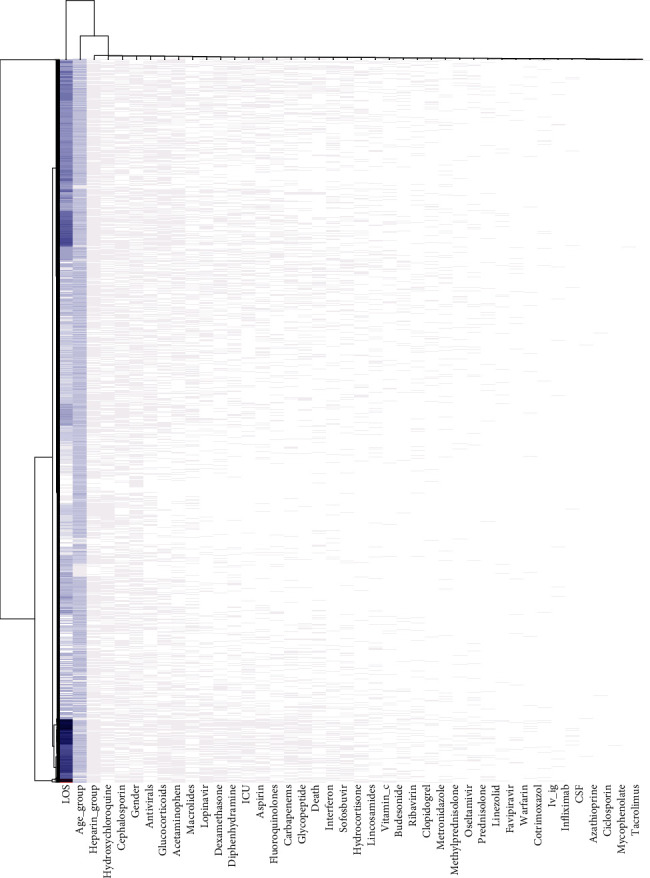
Data clustering level of our data mining study (for age group, the groups are 0-15, 16-30, 31-50, 51-80, and > 80).

All the individual data was clustered as a heat map to classify the patients based on their variables (Figure 3).

### 2.6. Data Analysis

To study univariate associations of the binary variables with death, the chi square test was used, and also relative risk (RR) and attributable risk (AR) were reported based on two by two cohort tables. To study univariate associations of the binary variables with LOS, the independent *t*-test was used. Bonferroni correction was applied on the *P* values. Regression modeling was used to adjust the effects the covariates. For death outcome, logistic regression was used reporting odds ratio (OR) with 95% confidence interval (CI). To study the performance of the model postestimation receiver operating characteristics (ROC) curve was used with reporting area under ROC curve (AUC). For LOS, right censored Poisson regression was used reporting incidence rate ratio (IRR) with 95% CI.

Although we were supposed to use stepwise method to remove the nuisance making covariates, we used the enter method instead, because of some reasons: (1) our dataset was too complex to decide about a specific model of the considered covariates, (2) some covariates were merely representative of some latent (unknown) variables, and we needed their adjusting effect rather than their own effects, and (3) since it was a preliminary report of this data mining study, we wanted to observe justice between all the covariates. Smith (2018) believed that the stepwise method might not be useful for big data [6]. Nevertheless, we designed a clinical modeling for hydroxychloroquine and another one for dexamethasone in which all the covariates were statistically significant. Dataset creation and data analysis were conducted in Stata 14 (StataCorp LLC, Texas, US) and *R* 3.6.3 (R foundation, Austria).

### 2.7. Ethical Considerations

The Ethics Committee of Iran University of Medical Sciences approved the protocol of study with registration number IR.IUMS.REC.1399.194.

## 3. Results

### 3.1. Primary Findings

**Figure 4:**
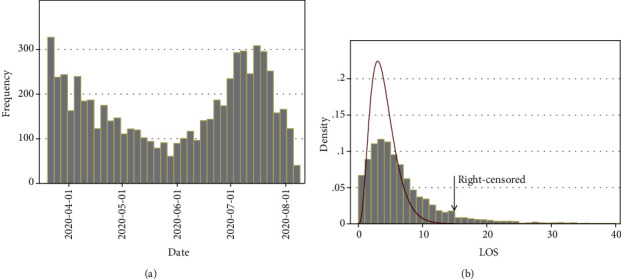
(a) Distribution of confirmed cases in our time frame. (b) Distribution of patients based on LOS and its comparison with the Poisson distribution.

A total of 6417 hospitalized confirmed cases were selected from 20 March 2020 to 11 August 2020 (Figure 4(a)). Among them, 61.2% were completely recovered, 13.8% were partially recovered, 19.3% were died, 4.5% were discharged by personal consent, 0.9% were transferred to another center, and 0.2% escaped from the hospital. The mean age was 56.89 ± 20.95 years, and 56% of the patients were male. About 27.3% of the patients were admitted to ICU. The mean of LOS was 6.23 ± 6.46 days (with interquartile range 2 - 8 days) (Figure 4(b)). Finally, 6054 cases remained for analysis after removing the cases discharged by personal consent, transferred to another center, and who escaped from the hospital. According to the association rule mining, age, ICU admission, glycopeptides, and carbapenems were associated with death, and ICU admission, glycopeptides, and carbapenems were associated with increased LOS (correlation coefficient > 0.2, *P* < 0.001) (Table 1). The most dominant and significant correlation was for the correlation of glycopeptides and carbapenems (Figures 1 and 2). All the cases were clustered according to their individual data. The most prevalent drugs were heparin_group, hydroxychloroquine and cephalosporins, respectively (Figure 3).

### 3.2. Associations with Death

Univariate wise, associations of the variables with death were studied in cohort tables. Accordingly, age ≥ 50 (RR = 3.10), ICU admission (RR = 2.99), carbapenems (RR = 2.90), glycopeptides (RR = 2.77), glucocorticoids (RR = 1.91), lincozamides (RR = 1.74), prednisolone (RR = 1.63), aspirin (RR = 1.54), dexamethasone (RR = 1.48), diphenhydramine (RR = 1.48), sofosbuvir/daclatasvir (RR = 1.47), metronidazole (RR = 1.47), clopidogrel (RR = 1.46), heparin_group (RR = 1.46), interferon (RR = 1.43), hydrocortisone (RR = 1.42), acetaminophen (RR = 1.40), lopinavir/ritonavir (RR = 1.41), fluoroquinolones (RR = 1.34), and antivirals (RR = 1.19) showed risk association, respectively, whereas cephalosporins (RR = 0.83) and macrolides (RR = 0.83) showed protecting association. No significant association was found for hydroxychloroquine (RR = 0.96) (Table 2).

**Figure 5:**
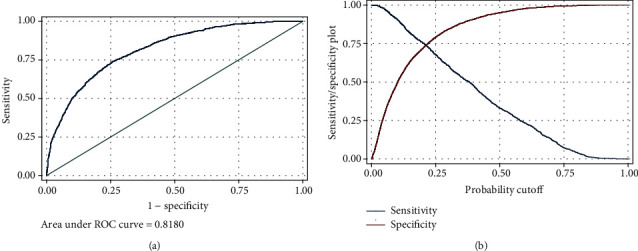
Performance of the logistic regression model (Table 3). The unit of the predictor is the result of the logistic function for each data as *y* = *β*1 × 1 + *β*2 × 2 + ⋯+*β*0. (a) ROC curve. (b) Sensitivity/spesificity plot.

Logistic regression was used to model the associations of the variables with death. Accordingly, age, male gender, ICU admission, prednisolone, lincosamides, glycopeptides, and carbapenems were risk predictors, whereas oseltamivir, cephalosporins, macrolides, and hydroxychloroquine were protecting predictors. At the next step, age interactions were added to the model. Accordingly, lopinavir became a risk predictor, and the protecting association of oseltamivir, macrolides, and hydroxychloroquine did not remain. At the final step, the interactions of ICU admission with the significant variables of the previous step were added to the model. Accordingly, the risk association of prednisolone was removed. None of the risk associations were more dominant than the association of ICU admission (considering ICU admission as a calibrator for lack of being a clinical risk factor in spite of being a risk predictor with OR = 6.26). The only protecting predictor of death was cephalosporins (OR = 0.45, 95%CI = 0.24 − 0.85, *P* = 0.015) (Table 3). The performance of the final step of this model for prediction of death is shown (AUC = 81.8%), and this model was more of specific rather than being sensitive (Figure 5).

**Figure 6:**
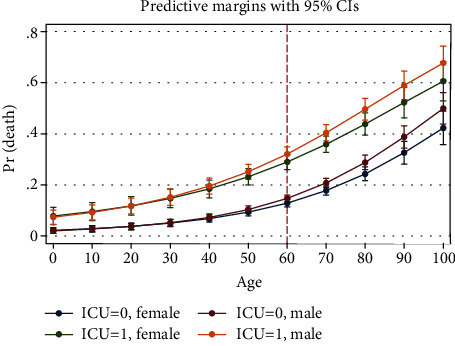
Association of gender with probability of death based on age and ICU admission (from logistic regression of Table 3, step 3).

**Figure 7:**
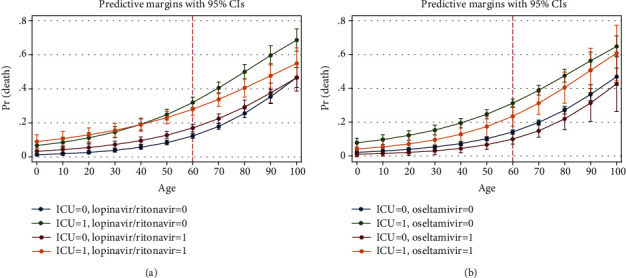
Association of lopinavir/ritonavir (a) and oseltamivir (b) with probability of death based on age and ICU admission (from logistic regression of Table 3, step 3).

**Figure 8:**
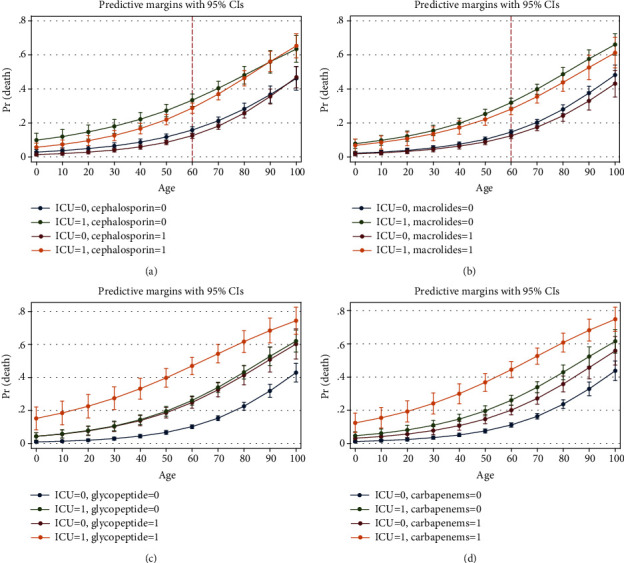
Association of cephalosporins (a), macrolides (b), glucopeptides (c), and carbapenems (d) with probability of death based on age and ICU admission (from logistic regression of Table 3, step 3).

**Figure 9:**
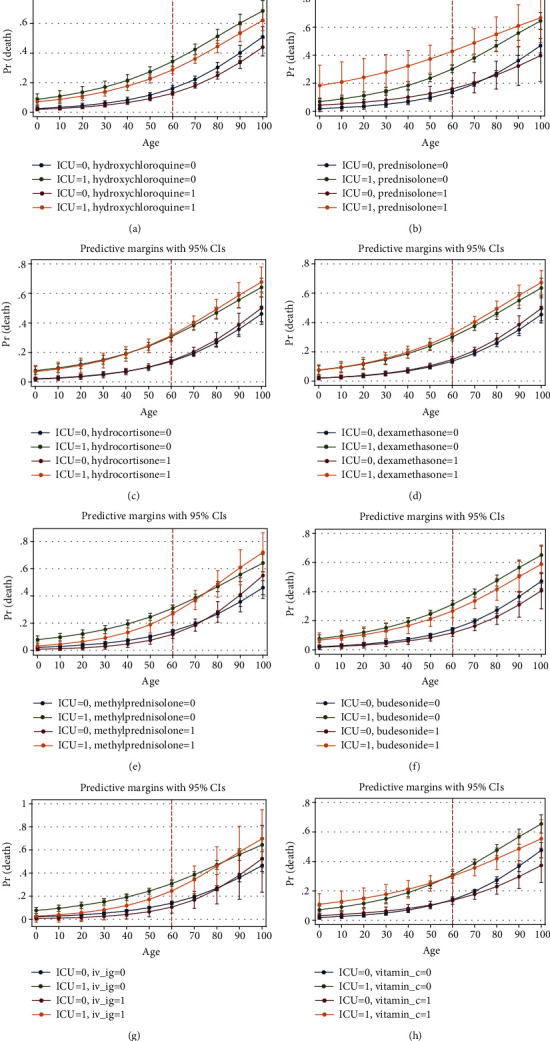
Association of hydroxichloroquine (a), prednisolon (b), hydrocortisone (c), dexamethasone (d), methylprednisolone (e), budesonide (f), IVIG (g), and vitamin C (h) with probability of death based on age and ICU admission (from logistic regression of Table 3, step 3).

Marginal analysis was performed on the final step of the logistic regression model to show the interactions of the variables with ICU admission at each age. After age 60, mortality in males was higher in both ICU admitted and unadmitted patients (Figure 6). Among the antivirals, lopinavir and oseltamivir were studied. Lopinavir was helpful in the ICU admitted patients after the age of 60. In patients without ICU admission, it might be harmful. Oseltamivir showed protecting effect in both ICU admitted and unadmitted patients (Figure 7). Among the antibiotics, cephalosporins were protective up to age 60 especially in ICU admitted patients. Protecting effect of macrolides was not dominant (Figure 8). Among the drugs of the immune system, hydroxychloroquine reduced mortality at most ages in both ICU admitted and unadmitted patients. Prednisolone was associated with increased mortality in ICU admitted patients. Hydrocortisone and dexamethasone showed no protecting or harmful effect. Methylprednisolone was helpful before the age of 60 in ICU admitted patients. Budesonide nebulizer seemed to be protecting but nonsignificant at many ages. IVIG was helpful by the age of 60 in ICU admitted patients. No significant association was found for vitamin C, but it might be helpful at ages more than 80 (Figure 9).

### 3.3. Modeling for Hydroxychloroquine

According to the controversial findings of hydroxychloroquine, a model was designed including age, ICU admission, hydroxychloroquine, underlying drugs (drugs for underlying cardiovascular or severe infectious diseases or drugs of diabetes), and their significant interactions. Accordingly, hydroxychloroquine showed a protecting effect before (OR = 0.79, 95%CI = 0.69 − 0.92, *P* < 0.001) and after (OR = 0.79, 95%CI = 0.69 − 0.92, *P* < 0.001) adjusting the interactions; however, interaction of receiving underlying drugs with hydroxychloroquine was significantly harmful (OR = 1.46, 95%CI = 1.06 − 2.03, *P* = 0.022) (Table 4).

### 3.4. Modeling for Dexamethasone

According to the controversial findings of dexamethasone, a model was designed including age, ICU admission, underlying drugs, underlying drugs (drugs for underlying cardiovascular or severe infectious diseases or drugs of diabetes), and their significant interactions. Accordingly, dexamethasone showed a harmful effect (OR = 1.18, 95%CI = 1.02 − 1.37, *P* = 0.025) (Table 5).

### 3.5. Associations with LOS

**Figure 10:**
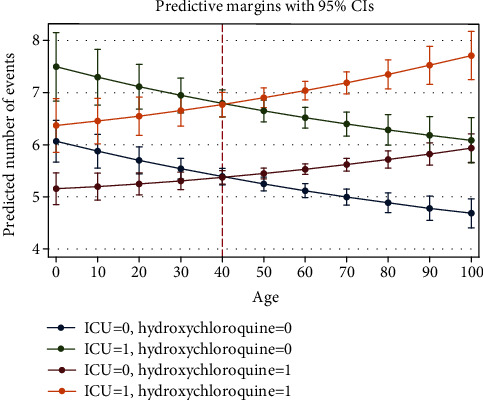
Association of hydroxychloroquine with LOS based on age and ICU admission (from Poisson regression of Table 4).

For this part, all the analyses were done on the improved cases. Univariate wise, associations of the variables with LOS were studied by the independent *t*-test. Most variables were associated with increased LOS. No variable was observed to decrease LOS significantly. Multivariable wise, right censored Poisson regression was used according to the distribution of LOS (Figure 4(b)). Among the variables, age and hydroxychloroquine were associated with decreased LOS (*P* < 0.001). Among the risk associations, ciclosporin and infliximab were more dominant than the association of ICU admission (as a calibrator for lack of being a risk factor instead of being a risk predictor with IRR = 1.24) (Table 6). Marginal analysis for interaction of the hydroxychloroquine and ICU admission at each age is shown. Accordingly, up to the age of 40 hydroxychloroquine was associated with decreased LOS and after the age of 40, it was associated with increased LOS in both ICU admitted and unadmitted patients (Figure 10).

## 4. Discussion

### 4.1. Summary of Evidence

According to the aims of our study, three main groups of drugs were investigated including antibiotics, antivirals, and drugs of the immune system. Of course, from the viewpoint of pharmacology, antibiotics and antivirals are chemotherapeutic agents and affect the immune system. We used regression modeling to adjust the confounding effects of the drugs. It seems that age was the most important comorbidity factor [7] and therefore, we adjusted its effect and its interactions in our models. Our association rule mining showed a rationale between the pairs of the variables, for example, the correlation of aspirin and clopidogrel indicating the patients with coronary artery disease. Other associations can also be detected (Figures 1 and 2). Individual data clustering showed that the most prevalent drug was the heparin_group—even more than hydroxychloroquine—due to our national protocol for anticoagulant therapy (Figure 3). For the associations of the dugs with the COVID-19 outcome, we discussed each drug separately. Before us, RECOVERY trial (randomized evaluation of COVID-19 therapy) had studied the effects of many of the drugs investigated in our study on thousands of participants.

#### 4.1.1. Sofosbuvir/Daclatasvir

In the cohort table, it was a risk factor for death. Regression wise, there was no association with death and LOS. It seems that its administration to complicated cases resulted in showing as risk factor in the cohort table. However, it seems that it does not have a beneficial effect. Unlike our study, an individual participant data meta-analysis showed that this drug could improve time to clinical recovery and reduce all causes of mortality. Many of the participants were Iranian [8]. Our association rule mining showed that its administration was usually along with dexamethasone indicating that our centers used it for more critical patients. It was not clear whether this combination was helpful for these patients or not.

#### 4.1.2. Lopinavir/Ritonavir (Kaletra)

In the cohort table, it was a risk factor. Regression wise, it was a risk factor for death after adjusting the interactions of age and ICU admission. However, its odds ratio was lower than ICU admission (2.84 vs 6.26) as the calibrator for the second generation null hypothesis (i.e., being clinically significant). Briefly, its effect was interaction-dependent. Marginal analysis showed a protecting effect for death in ICU admitted patients with age more than 60. It was a new finding in this study; however, its side effects and drug-drug interactions should be regarded for using in such patients [9]. Some meta-analyses did not show a beneficial effect [10, 11]. Our association rule mining showed that its administration was usually along with diphenhydramine and glucocorticoids mostly in ICU admitted patients with longer hospitalization.

#### 4.1.3. Ribavirin

In the cohort table, it did not show a significant association after bonferroni correction. Regression wise, no significant association was found for death and LOS. Our association rule mining showed that its administration was usually along with oseltamivir.

#### 4.1.4. Favipiravir

In the cohort table, it did not show a significant association after bonferroni correction. Regression wise, there was no association with death and LOS. A meta-analysis showed that patients had clinical and radiological improvements after treatment with favipiravir in comparison to standard care though no significant difference was observed for viral clearance and oxygen support [12].

#### 4.1.5. Oseltamivir

In the cohort table, it did not show a significant association after bonferroni correction. Regression wise, there was no association with death and LOS. Of course, in step 1 of our logistic regression analysis, it showed a protective association. According to the marginal analysis, its protecting association was more dominant in ICU admitted patients. There was not enough study about the effectiveness of this drug. Wu et al. showed that it might increase the survival rate in combination with lopinavir/ritonavir [13]. Our association rule mining showed that its administration was usually along with Ribavarin. Maybe some patients had received this drug for influenza prophylaxis due to their underlying conditions. However, it was the most effective antiviral agent in our study.

#### 4.1.6. Metronidazole

In the cohort table, it was a risk factor. Regression wise, there was no association with death and LOS. It seems that its administration to complicated cases resulted in showing as risk factor in the cohort table. Our association rule mining showed that its administration was high in patients with immunosuppressive drugs including ciclosporin, mycophenolate, and tacrolimus. We found no clinical study; however, some researchers considered a potential beneficial effect because of its anti-inflammatory effect and decreasing neutrophil count [14].

#### 4.1.7. Linezolid

In the cohort table, it did not show a significant association after bonferroni correction. Regression wise, there was no association with death and LOS. There was not a related clinical study. Our association rule mining showed that its administration was high in the patients receiving IVIG.

#### 4.1.8. Cephalosporins

In the cohort table, they were protecting factors. Regression wise, they were protecting factors for death while there was no effect on LOS. Marginal analysis showed that this protecting association was more dominant in younger ICU admitted patients. Our association rule mining showed that its administration was correlated with macrolides. It was not clear whether the protecting association of cephalosporins was due to its correlation with macrolide administration or due to its administration to more simple cases (the bias of selection by indication) or due to its own effect. In general, the associations of antibiotics with COVID-19 outcome are discussed with two aims: one of them is their effect on bacterial coinfections, and the second one is their antiviral effect. A computational analysis showed that chephalosporins had a potential binding ability to SARS-Cov-2 [15]. If an antibiotic shows a harmful effect, it may be representative of the effect of an underlying bacterial infection. Hence, if an antibiotic shows a protecting effect, this protecting effect is more reliable than that the harmful effect. In other words, it shows that these antibiotics have beneficial effect in spite of the underlying bacterial disease of the patients.

#### 4.1.9. Macrolides

In the cohort table, it was a protecting factor. Regression wise, there was no association with death and LOS. Of course, in step 1 of our logistic regression analysis, it showed a protective association. This possible protecting association was more dominant in older patients but similar response was observed in ICU admitted and unadmitted patients. Since macrolides increase QT interval, its contraindications should be regarded. A meta-analysis showed that azithromycin increased mortality in combination with hydroxychloroquine [16].

#### 4.1.10. Fluoroquinolones

In the cohort table, it was a risk factor. Regression wise, it was a risk factor for LOS. Our association rule mining showed that its administration was correlated with administration of hydrocortisone.

#### 4.1.11. Lincosamides

In the cohort table, it was a risk factor. Regression wise, there was no association with death and LOS.

#### 4.1.12. Glycopeptides

In the cohort table, this group of anibiotics was a risk factor. Regression wise, it was a risk factor for death and LOS. Our association rule mining showed that its administration was usually along with carbapenems. Its odds ratio was not significantly lower than the odds ratio of ICU admission (5.38 vs 6.26) as the calibrator for the second generation null hypothesis (i.e., being clinically significant). It means that it can show severity of the underlying infectious comorbidity like ICU admission shows severity of the disease. Previous studies have shown that bacterial coinfection might occur in severe cases of COVID-19. This coinfection is not easy to follow because the inflammatory biomarkers are not specific for it [17, 18].

#### 4.1.13. Carbapenems

Their effect was approximately similar to glycopeptides as discussed above.

#### 4.1.14. Cotrimoxazole

In the cohort table, it was not a risk factor. Regression wise, there was no association with death and LOS. Our association rule mining showed that its administration was correlated with azathioprine.

#### 4.1.15. Interferon

In the cohort table, it was a risk factor. Regression wise, there was no association with death, and there was no association with LOS. Its risk association in the cohort table might be due to its administration to more sever patients. Our association rule mining showed that its administration was correlated with acetaminophen.

#### 4.1.16. Dexamethasone

In the cohort table, it was a risk factor. Regression wise, there was no association with death and LOS. In a meta-analysis of clinical trials of critically ill patients with COVID-19, administration of systemic corticosteroids (dexamethasone), compared with usual care or placebo, was associated with lower 28-day all-cause mortality [19]. Nevertheless, its routine administration is not recommended according to our results (marginal analysis did not show a protecting effect). Our association rule mining showed that its administration was correlated with sofosbuvir. According to the controversies, we decided to design a practical model. Although based on this model, dexamethasone showed a harmful effect (Table 5), its odds ratio was lower than the odds ratio of ICU admission. Based on the results of the RECOVERY collaborative group, only patients with mechanical ventilation benefit from dexamethasone [20].

#### 4.1.17. Hydrocortisone

In the cohort table, it was a risk factor for death. Regression wise, there was no association with death and LOS. Our association rule mining showed that its administration was correlated with fluoroquinolone. Marginal analysis did not show a protecting effect.

#### 4.1.18. Budesonide

In the cohort table, it did not show a significant association after bonferroni correction. Regression wise, there was no association with death and LOS. Our association rule mining showed that its administration was correlated with sofosbuvir and dexamethasone. Marginal analysis did not show a protecting affect.

#### 4.1.19. Prednisolone

In the cohort table, it was a risk factor for death. Regression wise, there was no association with LOS. According to our logistic regression, it was a risk factor for death, but this association was removed after adjusting the interactions of ICU admission. In a systematic review of 89 studies, administration of low-dose prednisolone had beneficial impacts on COVID-19 [21]. However, it seems that its administration without an indication other than COVID-19 may be harmful according to our results. Our association rule mining showed that its administration was correlated with death, glycopeptides, and carbapenems. It showed that its administration was common in patients with underlying infectious diseases.

#### 4.1.20. Methylprednisolone

In the cohort table, it did not show a significant association after bonferroni correction. Regression wise, it was associated with increased LOS after adjusting the interactions of age and ICU admission, but there was no association with death. Our association rule mining showed that its administration was correlated with ribavirin and oseltamivir. Although our results could not show a significant protecting effect on death, it seemed that methylprednisolone pulse might be useful in critically ill patients according to the literature [22, 23]. Marginal analysis showed that it was protective in ICU admitted patients with lower ages.

#### 4.1.21. Infliximab

In the cohort table, it did not show a significant association after bonferroni correction. Regression wise, it was associated with increased LOS after adjusting the interactions of age and ICU admission, but there was no association with death. Our association rule mining showed that it was in the cluster of immunosuppressive agents (Figure 2, right cluster).

#### 4.1.22. Mycophenolate

There was not enough observation to judge. Our association rule mining showed that its administration was correlated with tacrolimus.

#### 4.1.23. IVIG

In the cohort table, it did not show a significant association after bonferroni correction. Regression wise, there was no association with death and LOS. A meta-analysis of two studies with total of 383 critical COVID-19 patients showed that administration of high-dose IVIG in first week of disease course especially in first 2 days was significantly associated with lower mortality and hospital stay rate [24]. Our association rule mining showed that it was in the cluster of immunosuppressive agents (Figure 2, right cluster).

#### 4.1.24. Vitamin C

In the cohort table, it did not show a significant association after bonferroni correction. Regression wise, there was no association with death but an increased in LOS was seen. Vitamin C has potentially antiviral effects through producing free radicals and rising antiviral cytokines; however, putting study design problems aside, not enough evidence for routine use of vitamin C in COVID-19 patients was found [25, 26]. Our association rule mining showed that its administration was usually along with favipiravir, interferon, and acetaminophen.

#### 4.1.25. Azathioprine

There was not enough observation to judge.

#### 4.1.26. Ciclosporin

There was not enough observation to judge.

#### 4.1.27. Tacrolimus

There was not enough observation to judge.

#### 4.1.28. CSF

There was not enough observation to judge.

#### 4.1.29. Hydroxychloroquine

The most controversial drug of this study was hydroxychloroquine. In the cohort table, no significant result was seen. In logistic regression, it showed protecting effect before adjusting the interactions. Marginal analysis showed a protecting effect on death for ages upper than about 50, and a protecting effect on LOS for ages lower than about 40. Nevertheless, literature did not support our results [27, 28]. In addition, the results of the RECOVERY collaborative group did not support a protecting effect for hydroxychloroquine [29]. According to the controversies, we decided to design a practical model. Using this drug in patients with diabetes or underlying cardiovascular or infectious diseases is not recommended. Its drug interactions about increasing QT interval should be regarded. However, its administration to other hospitalized patients may be helpful according to our results.

#### 4.1.30. Aspirin

In the cohort table, it was a risk factor for death. Regression wise, there was no association with death but an increase in LOS was seen. Our association rule mining showed that its administration was correlated with clopidogrel showing cardiovascular disease in such patients.

#### 4.1.31. Warfarin

In the cohort table, it did not show a significant association after bonferroni correction. Regression wise, there was no association with death and LOS . Our association rule mining showed that it was in the cluster of immunosuppressive agents (Figure 2, right cluster).

#### 4.1.32. Clopidogrel

Our association rule mining showed that its administration was correlated with aspirin showing cardiovascular disease in such patients.

#### 4.1.33. Heparins

In the cohort table, it was a risk factor for death. Regression wise, there was no association with death and LOS. In a systematic review of 11 studies, administration preventive doses of anticoagulants, including low-molecular weight heparins or unfractionated heparin were indicated to all hospitalized patients with COVID-19 and had beneficial impacts on them [30]. Although we did not find a beneficial effect, it may be necessary for prevention of pulmonary thromboembolism.

#### 4.1.34. Acetaminophen

In the cohort table, it was a risk factor for death. Regression wise, there was no association with death but an increase in LOS was seen. Our association rule mining showed that its administration was correlated with interferon, vitamin C, and favipiravir.

#### 4.1.35. Diphenhydramine

In the cohort table, it was a risk factor for death. Regression wise, there was no association with death but an increase in LOS was seen. Our association rule mining showed that its administration was correlated with lopinavir/ritonavir, glucocorticoids, LOS, and ICU.

### 4.2. Limitations

It was a primary report of a complex dataset. Therefore, this article did not have an enough space for subgroup and more specific analyses. In addition, in a data mining study, the patients, the exposures, and the interventions are not based on the design of the researcher but all of them are based on the existed indications. In the case of ours, many drugs were not clinical trial administration but they were used for underlying indications of the patients. In other words, we had a bias called selection by indication (selection bias confounded by indication). This bias resulted that some simple drugs showed protecting association due to administration to simple cases, and some more specific drugs showed risk association due to administration to complex cases. Regression modeling could help us to overcome these limitations. Nevertheless, our models were complex with some nuisance making covariates. However, the stepwise method showed similar results (tables not shown). Being retrospective was another limitation in our study. Prospective studies and well-designed clinical trials should be performed regarding the effective covariates and interactions such as ICU admission and underlying diseases.

## 5. Conclusions

As a data mining study, it was not conclusive whether the protecting or risk associations were due to causation or they were confounded by bias in case selection for each drug. We could not reach a model for exact prediction of the outcome. Association of hydroxychloroquine with COVID-19 outcome was controversial. Although hydroxychloroquine showed a protecting effect in our regression modeling of all the drugs, this association did not remain after adjusting the interactions. It seems that the associations of hydroxychloroquine and other drugs are correlated with some known and unknown interactions. Administration corticosteroids should be based on the conditions of each case. Protective effects of cephalosporins was another notable finding. The data of this study was for the time when using remdesivir was not common and available in our country and also we had extra ICU mortality in the mentioned period of time. As a preliminary report, the results of this study should be used for hypothesis creation for clinical trials or hypothesis creation for more specific data mining studies. The approach and methodology of this study is suggested to be used for other diseases and health issues.

## Figures and Tables

**Table 1:** Association rule mining of all variables with death and LOS.

Variable	Frequency (%)	Association with death				Association with LOS	
		Support (variable⟶death)	Confidence (variable⟶death)	Correlation coefficient	Bonferroni adjusted *P* value	Correlation coefficient	Bonferroni adjusted *P* value
Total COVID-19 cases	6417 (100)	0.193	0.193				
Death	1240 (19.32)	0.193	1	1.000	1.000	0.075	<0.001^∗∗∗∗^
LOS (mean ± SD)	6.23 ± 6.46			0.075	<0.001^∗∗∗∗^	1.000	1.000
Age (mean ± SD)	56.89 ± 20.95			0.258#	<0.001^∗∗∗∗^	0.036	1.000
Gender (male)	3597 (56.05)	0.113	0.202	0.026	1.000	0.014	1.000
ICU	1751 (27.29)	0.104	0.380	0.289#	<0.001^∗∗∗∗^	0.337#	<0.001^∗∗∗∗^
Date (range, in 2020)	03-20–08-11			-0.011	1.000	-0.103	<0.001^∗∗∗∗^
Sofosbuvir/daclatasvir	1116 (17.39)	0.047	0.269	0.088	<0.001^∗∗∗∗^	0.242#	<0.001^∗∗∗∗^
Lopinavir/ritonavir	1962 (30.58)	0.075	0.247	0.090	<0.001^∗∗∗∗^	0.269#	<0.001^∗∗∗∗^
Ribavirin	530 (8.26)	0.020	0.254	0.040	1.000	0.174	<0.001^∗∗∗∗^
Favipiravir	155 (2.42)	0.007	0.277	0.034	1.000	0.087	<0.001^∗∗∗∗^
Oseltamivir	342 (5.33)	0.011	0.208	0.009	1.000	0.134	<0.001^∗∗∗∗^
Interferon	1145 (17.84)	0.046	0.259	0.078	<0.001^∗∗∗∗^	0.239	<0.001^∗∗∗∗^
Metronidazole	347 (5.41)	0.015	0.282	0.054	0.014^∗^	0.133	<0.001^∗∗∗∗^
Linezolid	159 (2.48)	0.007	0.283	0.036	1.000	0.125	<0.001^∗∗∗∗^
Dexamethasone	1817 (28.32)	0.072	0.255	0.098	<0.001^∗∗∗∗^	0.293#	<0.001^∗∗∗∗^
Hydrocortisone	861 (13.42)	0.053	0.264	0.070	<0.001^∗∗∗∗^	0.246#	<0.001^∗∗∗∗^
Budesonide	564 (8.79	0.021	0.241	0.038	1.000	0.183	<0.001^∗∗∗∗^
Prednisolone	275 (4.29)	0.013	0.309	0.062	<0.001^∗∗∗∗^	0.118	<0.001^∗∗∗∗^
Methylprednisolone	349 (5.44)	0.012	0.229	0.022	1.000	0.155	<0.001^∗∗∗∗^
Infliximab	86 (1.34)	0.004	0.302	0.032	1.000	0.096	<0.001^∗∗∗∗^
Mycophenolate	10 (0.16)	0.000	0.200	0.001	1.000	0.063	<0.001^∗∗∗^
IV Ig	94 (1.46)	0.004	0.277	0.026	1.000	0.085	<0.001^∗∗∗∗^
Vitamin C	601 (9.37)	0.022	0.236	0.035	1.000	0.179	<0.001^∗∗∗∗^
Azathioprine	10 (0.16)	0.000	0.300	0.011	1.000	-0.012	1.000
Ciclosporin	4 (0.06)	0.000	0.500	0.019	1.000	0.018	1.000
Tacrolimus	7 (0.11)	0.000	0.286	0.008	1.000	<0.001	1.000
Aspirin	1611 (25.11)	0.067	0.276	0.108	<0.001^∗∗∗∗^	0.290#	<0.001^∗∗∗∗^
Warfarin	153 (2.38)	0.006	0.261	0.027	1.000	0.110	<0.001^∗∗∗∗^
Clopidogrel	490 (7.64)	0.021	0.278	0.061	<0.001^∗∗∗^	0.172	<0.001^∗∗∗∗^
Acetaminophen	2897 (45.15)	0.105	0.232	0.089	<0.001^∗∗∗∗^	0.296#	<0.001^∗∗∗∗^
Diphenhydramine	1736 (27.05)	0.070	0.257	0.098	<0.001^∗∗∗∗^	0.258#	<0.001^∗∗∗∗^
Antivirals	3135 (48.85)	0.105	0.215	0.053	0.0191^∗^	0.118	<0.001^∗∗∗∗^
CSF	21 (0.38)	0.001	0.238	0.007	1.000	0.061	0.001^∗∗^
Cephalospoins	3715 (57.89)	0.103	0.178	-0.046	0.243	0.067	<0.001^∗∗∗^
Macrolides	2004 (31.23)	0.054	0.172	-0.035	1.000	0.035	1.000
Fluoroquinolones	1509 (23.52)	0.057	0.245	0.073	<0.001^∗∗∗∗^	0.283#	<0.001^∗∗∗∗^
Lincosamides	766 (11.94)	0.038	0.319	0.117	<0.001^∗∗∗∗^	0.119	<0.001^∗∗∗∗^
Glycopeptides	1383 (21.55)	0.084	0.389	0.260#	<0.001^∗∗∗∗^	0.318#	<0.001^∗∗∗∗^
Carbapenems	1537 (23.95)	0.093	0.389	0.279#	<0.001^∗∗∗∗^	0.333#	<0.001^∗∗∗∗^
Cotrimoxazol	101 (1.57)	0.005	0.287	0.030	1.000	0.091	<0.001^∗∗∗∗^
Glucocorticoids	3022 (47.09)	0.123	0.261	0.161	<0.001^∗∗∗∗^	0.300#	<0.001^∗∗∗∗^
Heparin group	5054 (78.76)	0.165	0.209	0.078	<0.001^∗∗∗∗^	0.232#	<0.001^∗∗∗∗^
Hydroxychloroquine	3997 (62.28)	0.121	0.194	0.004	1.000	0.089	<0.001^∗∗∗∗^

^∗^Significant at 0.05. ^∗∗^Significant at 0.01. ^∗∗∗^Significant at 0.001. ^∗∗∗∗^Significant at 0.0001. #Correlation coefficient more than 0.2. All the *P* values are for correlation analysis.

**Table 2:** Association of the variables with risk of death (in hospitalized improved or dead cases, the other cases were removed).

Variables	Relative risk		Attributable risk		Chi square
	Point estimation	95% CI	Point estimation	95% CI	*P* value
Age ≥ 50	3.10	2.65, 3.61^∗^	0.18	0.16, 0.20^∗^	<0.001^∗∗^
ICU	2.99	2.71, 3.29^∗^	0.26	0.24, 0.29^∗^	<0.001^∗∗^
Sofosbuvir/daclatasvir	1.47	1.31, 1.65^∗^	0.09	0.06, 0.12^∗^	<0.001^∗∗^
Lopinavir/ritonavir	1.41	1.28, 1.56^∗^	0.08	0.05, 0.10^∗^	<0.001^∗∗^
Ribavirin	1.27	1.08, 1.48^∗^	0.05	0.01, 0.09^∗^	0.004
Favipiravir	1.44	1.12, 1.87^∗^	0.09	0.02, 0.16^∗^	0.008
Oseltamivir	1.06	0.85, 1.31	0.01	-0.03, 0.06	0.612
Interferon	1.43	1.28, 1.60^∗^	0.08	0.05, 0.11^∗^	<0.001^∗∗^
Metronidazole	1.47	1.23, 1.75^∗^	0.09	0.04, 0.14^∗^	<0.001^∗∗^
Linezolid	1.47	1.15, 1.89^∗^	0.10	0.02, 0.17^∗^	0.004
Dexamethasone	1.48	1.34, 1.64^∗^	0.09	0.06, 0.11^∗^	<0.001^∗∗^
Hydrocortisone	1.42	1.25, 1.61^∗^	0.08	0.05, 0.11^∗^	<0.001^∗∗^
Budesonide	1.26	1.08, 1.47^∗^	0.05	0.014, 0.09^∗^	0.004
Prednisolone	1.63	1.36, 1.95^∗^	0.13	0.07, 0.18^∗^	<0.001^∗∗^
Methylprednisolone	1.18	0.97, 1.44	0.04	-0.01, 0.08	0.106
Infliximab	1.54	1.12, 2.13^∗^	0.11	0.01, 0.21^∗^	0.014
Mycophenolate	0.98	0.28, 3.38	-0.00	-0.25, 0.24	0.970
IV_Ig	1.39	1.00, 1.93	0.08	-0.01, 0.17	0.063
Vitamin C	1.22	1.05, 1.42^∗^	0.04	0.01, 0.08^∗^	0.013
Azathioprine	1.47	0.57, 3.78	0.10	-0.19, 0.38	0.455
Ciclosporin	2.44	0.92, 6.52	0.30	-0.19, 0.79	0.143
Tacrolimus	1.40	0.43, 4.51	0.08	-0.25, 0.42	0.596
Aspirin	1.54	1.39, 1.70^∗^	0.10	0.07, 0.12^∗^	<0.001^∗∗^
Warfarin	1.31	1.00, 1.72	0.06	-0.09, 0.13	0.057
Clopidogrel	1.46	1.26, 1.70^∗^	0.09	0.05, 0.13^∗^	<0.001^∗∗^
Acetaminophen	1.40	1.27, 1.55^∗^	0.07	0.05, 0.09^∗^	<0.001^∗∗^
Diphenhydramine	1.48	1.34, 1.64^∗^	0.09	0.06, 0.11^∗^	<0.001^∗∗^
Antivirals	1.19	1.08, 1.32^∗^	0.04	0.02, 0.06^∗^	<0.001^∗∗^
CSF	1.22	0.57, 2.61	0.05	-0.14, 0.24	0.616
Cephalosporins	0.83	0.75, 0.92	-0.04	-0.06, -0.02^∗^	<0.001^∗∗^
Macrolides	0.83	0.75, 0.93	-0.04	^-0.06, -0.01^^∗^	^0.001^^∗∗^
Fluoroquinolones	1.34	1.21, 1.49^∗^	0.06	0.04, 0.09^∗^	<0.001^∗∗^
Lincosamides	1.74	1.55, 1.96^∗^	0.14	0.10, 0.17^∗^	<0.001^∗∗^
Glycopeptides	2.77	2.52, 3.04^∗^	0.26	0.23, 0.29^∗^	<0.001^∗∗^
Carbapenems	2.90	2.64, 3.19^∗^	0.27	0.24, 0.29^∗^	<0.001^∗∗^
Cotrimoxazole	1.46	1.07, 1.98^∗^	0.09	0.00,0.18^∗^	0.024
Glucocorticoids	1.91	1.72, 2.12^∗^	0.13	0.11, 0.15^∗^	<0.001^∗∗^
Heparin_group	1.46	1.26, 1.68^∗^	0.07	0.05, 0.09^∗^	<0.001^∗∗^
Hydroxychloroquine	0.96	0.87, 1.06	-0.01	-0.03, 0.01	0.428

^∗^Significant at H0: RR = 1 or AR = 0. ^∗∗^Significant at 0.0013 (Pearson chi square, according to bonferroni correction).

**Table 3:** Logistic regression model for association of the variables with odds of death (in hospitalized improved or dead cases, the other cases were removed).

Model	Step 1: adjusted with each other		Step 2: step 1 plus adjustment with age interactions		Step 3: step 2 plus adjustment with some ICU interactions	
Covariates	Adjusted odds ratio (95% CI)	*P* value	Adjusted odds ratio (95% CI)	*P* value	Adjusted odds ratio (95% CI)	*P* value
Age	1.041 (1.036, 1.045)	<0.001^∗^	1.047 (1.033, 1.062)	<0.001^∗^	1.048 (1.033, 1.063)	<0.001^∗^
Gender (male)	1.212 (1.048, 1.402)	0.010^∗^	0.896 (0.492, 1.632)	0.720	0.917 (0.503, 1.673)	0.778
Date (daily)	1.001 (0.999, 1.003)	0.377	1.001 (0.999, 1.003)	0.356	1.001 (0.999, 1.003)	0.372
ICU	2.919 (2.502, 3.405)	0.000^∗^	4.855 (2.582, 9.130)	<0.001^∗^	6.257 (3.142, 12.46)	<0.001^∗^
Sofosbuvir/daclatasvir	1.081 (0.891, 1.310)	0.430	1.696 (0.796, 3.610)	0.171	1.755 (0.824, 3.738)	0.145
Lopinavir/ritonavir	1.080 (0.913, 1.276)	0.370	2.031 (1.047, 3.943)	0.036^∗^	2.837 (1.442, 5.581)	0.003^∗^
Ribavirin	0.976 (0.756, 1.261)	0.855	0.539 (0.196, 1.479)	0.230	0.541 (0.198, 1.476)	0.230
Favipiravir	0.985 (0.636, 1.524)	0.945	0.215 (0.026, 1.791)	0.155	0.221 (0.027, 1.840)	0.163
Oseltamivir	0.628 (0.451, 0.873)	0.006^∗^	0.445 (0.109, 1.823)	0.261	0.433 (0.106, 1.761)	0.242
Interferon	1.014 (0.837, 1.230)	0.884	0.696 (0.319, 1.515)	0.361	0.689 (0.316, 1.500)	0.348
Metronidazole	1.077 (0.804, 1.443)	0.620	1.331 (0.377, 4.697)	0.657	1.338 (0.379, 4.729)	0.651
Linezolid	1.083 (0.705, 1.665)	0.715	0.452 (0.074, 2.747)	0.388	0.472 (0.078, 2.871)	0.415
Dexamethasone	1.099 (0.924, 1.306)	0.287	1.057 (0.519, 2.154)	0.879	1.005 (0.493, 2.048)	0.989
Hydrocortisone	1.083 (0.882, 1.330)	0.445	0.837 (0.359, 1.952)	0.681	0.870 (0.374, 2.024)	0.746
Budesonide	0.778 (0.605, 1.001)	0.051	0.812 (0.302, 2.182)	0.680	0.837 (0.313, 2.241)	0.723
Prednisolone	1.411 (1.027, 1.940)	0.034^∗^	3.448 (1.007, 11.807)	0.049^∗^	2.640 (0.703, 9.923)	0.151
Methylprednisolone	0.891 (0.652, 1.218)	0.469	0.291 (0.074, 1.140)	0.076	0.316 (0.081, 1.234)	0.097
Infliximab	1.445 (0.830, 2.516)	0.193	3.58 (0.537, 23.857)	0.188	3.703 (0.556, 24.669)	0.176
Mycophenolate	0.195 (0.022, 1.746)	0.144	NA		NA	NA
IV_Ig	0.779 (0.447, 1.359)	0.379	0.280 (0.028, 2.842)	0.282	0.278 (0.028, 2.736)	0.272
Vitamin C	0.904 (0.710, 1.151)	0.412	1.720 (0.677, 4.369)	0.254	1.793 (0.706, 4.553)	0.220
Azathioprine	1.623 (0.328, 8.040)	0.553	NA		NA	NA
Ciclosporin	3.91 (0.356, 42.909)	0.265	NA		NA	NA
Tacrolimus	3.687 (0.387, 35.08)	0.256	NA		NA	NA
Aspirin	1.094 (0.919, 1.304)	0.312	1.254 (0.627, 2.510)	0.522	1.253 (0.625, 2.511)	0.525
Warfarin	1.012 (0.653, 1.567)	0.959	1.135 (0.227, 5.668)	0.878	1.128 (0.227, 5.605)	0.883
Clopidogrel	0.956 (0.734, 1.245)	0.738	0.792 (0.267, 2.344)	0.673	0.770 (0.260, 2.278)	0.636
Acetaminophen	0.982 (0.836, 1.155)	0.830	0.909 (0.464, 1.780)	0.781	0.880 (0.449, 1.726)	0.711
Diphenhydramine	1.161 (0.980, 1.376)	0.085	1.385 (0.712, 2.693)	0.338	1.382 (0.712, 2.685)	0.339
CSF	1.363 (0.402, 4.618)	0.619	4.567 (0.535, 38.971)	0.165	4.613 (0.542, 39.237)	0.162
Cephalosporins	0.794 (0.680, 0.927)	0.003^∗^	0.451 (0.243, 0.839)	0.012^∗^	0.447 (0.235, 0.853)	0.015^∗^
Macrolides	0.825 (0.699, 0.972)	0.022^∗^	0.857 (0.431, 1.703)	0.659	0.843 (0.423, 1.683)	0.628
Fluoroquinolones	0.893 (0.751, 1.061)	0.199	1.011 (0.508, 2.013)	0.976	1.047 (0.526, 2.084)	0.897
Lincosamides	1.811 (1.482, 2.213)	<0.001^∗^	1.749 (0.771, 3.970)	0.181	1.714 (0.754, 3.895)	0.198
Glycopeptide	2.811 (2.358, 3.350)	<0.001^∗^	5.012 (2.505, 10.026)	<0.001^∗^	5.377 (2.617, 11.047)	<0.001^∗^
Carbapenems	2.141 (1.806, 2.537)	<0.001^∗^	3.181 (1.574, 6.430)	0.001^∗^	2.935 (1.406, 6.124)	0.004^∗^
Cotrimoxazol	0.748 (0.440, 1.272)	0.284	0.776 (0.068, 8.801)	0.838	0.788 (0.070, 8.840)	0.847
Heparin_group	0.882 (0.718, 1.084)	0.233	0.873 (0.397, 1.920)	0.736	0.868 (0.393, 1.918)	0.726
Hydroxychloroquine	0.735 (0.627, 0.862)	<0.001^∗^	0.757 (0.410, 1.398)	0.373	0.751 (0.406, 1.388)	0.361

The additional interactions of step 3 are the interactions of ICU admission with the significant results of step 2. NA: not applicable due to low number of observations. ^∗^Significant at 0.05.

**Table 4:** Logistic regression model for association of hydroxychloroquine with death and the significant interactions.

Model	Step 1: adjusted with each other		Step 2: step 1 plus interactions	
Covariates	Adjusted odds ratio (95% CI)	*P* value	Adjusted odds ratio (95% CI)	*P* value
Age	1.038 (1.034, 1.042)	<0.001^∗^	1.044 (1.038, 1.050)	<0.001^∗^
ICU	3.428 (2.981, 3.941)	<0.001^∗^	7.590 (4.354, 13.233)	<0.001^∗^
Underlying drugs	2.348 (1.996, 2.762)	<0.001^∗^	1.869 (1.461, 2.391)	<0.001^∗^
Hydroxychloroquine (HCQ)	0.794 (0.689, 0.916)	0.002^∗^	0.600 (0.453, 0.794)	<0.001^∗^
Significant interactions				
Age #ICU			0.988 (0.980, 0.996)	0.004^∗^
Underlying drugs #HCQ			1.464 (1.057, 2.029)	0.022^∗^

^∗^Significant at 0.05. #Interaction sign.

**Table 5:** Logistic regression model for association of dexamethasone with death and the significant interactions.

Model	Step 1: adjusted with each other		Step 2: step 1 plus interactions	
Covariates	Adjusted odds ratio (95% CI)	*P* value	Adjusted odds ratio (95% CI)	*P* value
Age	1.038 (1.034, 1.042)	<0.001^∗^	1.044 (1.038, 1.050)	<0.001^∗^
ICU	3.335 (2.898, 3.838)	<0.001^∗^	7.448 (4.266, 13.005)	<0.001^∗^
Underlying drugs	2.259 (1.919, 2.660)	<0.001^∗^	2.245 (1.907, 2.643)	<0.001^∗^
Dexamethasone	1.181 (1.020, 1.368)	0.026^∗^	1.183 (1.022, 1.369)	0.025^∗^
Significant interactions				
Age #ICU			0.988 (0.980, 0.996)	0.003^∗^
Interaction not assumed				
ICU #dexamethasone			0.795 (0.595, 1.060)	0.119

^∗^Significant at 0.05. #Interaction sign.

**Table 6:** Association of the variables with LOS in survived cases using the independent *t*-test and right censored Poisson regression (in hospitalized improved cases; the other cases were removed).

Analysis	*t*-test		Right censored Poisson regression (upper limit: LOS = 15)	
Covariates	Mean difference	*P* value	Adjusted incidence rate ratio (95% CI)	*P* value
Age ≥50	0.673	<0.001^∗∗^	0.994 (0.992, 0.996)	<0.001^∗^
Gender (male)	-0.097	0.580	1.066 (0.991, 1.146)	0.088
Date (daily)	NA	NA	1.000 (1.000, 1.000)	0.590
ICU	5.124	<0.001^∗∗^	1.235 (1.129, 1.352)	<0.001^∗^
Sofosbuvir/daclatasvir	3.713	<0.001∗∗	1.059 (0.958, 1.172)	0.263
Lopinavir/ritonavir	3.580	<0.001^∗∗^	1.073 (0.985, 1.168)	0.107
Ribavirin	4.061	<0.001^∗∗^	1.155 (1.013, 1.315)	0.031^∗^
Favipiravir	2.926	<0.001^∗∗^	1.034 (0.799, 1.337)	0.801
Oseltamivir	3.769	<0.001^∗∗^	1.042 (0.882, 1.231)	0.628
Interferon	3.960	<0.001^∗∗^	1.021 (0.925, 1.127)	0.680
Metronidazole	2.577	<0.001^∗∗^	1.038 (0.875, 1.231)	0.672
Linezolid	4.653	<0.001^∗∗^	1.087 (0.858, 1.379)	0.488
Dexamethasone	3.827	<0.001^∗∗^	1.206 (1.102, 1.320)	<0.001^∗^
Hydrocortisone	3.974	<0.001^∗∗^	1.072 (0.961, 1.195)	0.213
Budesonide	3.924	<0.001^∗∗^	0.936 (0.828, 1.059)	0.293
Prednisolone	2.858	<0.001^∗∗^	1.070 (0.851, 1.345)	0.562
Methylprednisolone	3.994	<0.001^∗∗^	1.311 (1.120, 1.534)	0.001^∗^
Infliximab	4.340	<0.001^∗∗^	1.800 (1.363, 2.378)	<0.001^∗^
Mycophenolate	0.941	0.659	0.552 (0.124, 2.464)	0.436
IV_Ig	4.176	<0.001^∗∗^	0.883 (0.655, 1.189)	0.411
Vitamin C	4.486	<0.001^∗∗^	1.231 (1.091, 1.390)	<0.001^∗^
Azathioprine	-0.472	0.836	0.487 (0.092, 2.565)	0.396
Ciclosporin	2.316	0.587	16.539 (1.42, 192.63)	0.025^∗^
Tacrolimus	-1.387	0.608	0.503 (0.049, 5.219)	0.565
Aspirin	3.705	<0.001^∗∗^	1.097 (1.001, 1.202)	0.047^∗^
Warfarin	4.276	<0.001^∗∗^	1.206 (0.962, 1.512)	0.104
Clopidogrel	3.737	<0.001^∗∗^	1.331 (1.145, 1.547)	<0.001^∗^
Acetaminophen	3.431	<0.001^∗∗^	1.205 (1.109, 1.308)	<0.001^∗^
Diphenhydramine	3.333	<0.001^∗∗^	1.114 (1.024, 1.213)	0.013^∗^
CSF	8.508	<0.001^∗∗^	1.563 (1.078, 2.265)	0.018^∗^
Cephalosporins	0.455	0.010	1.067 (0.987, 1.154)	0.103
Macrolides	0.022	0.905	0.922 (0.847, 1.004)	0.063
Fluoroquinolones	4.031	<0.001^∗∗^	1.110 (1.018, 1.210)	0.018^∗^
Lincosamides	1.933	<0.001^∗∗^	1.056 (0.932, 1.195)	0.393
Glycopeptide	5.447	<0.001^∗∗^	1.422 (1.292, 1.566)	<0.001^∗^
Carbapenems	5.271	<0.001^∗∗^	1.298 (1.174, 1.435)	<0.001^∗^
Cotrimoxazol	3.481	<0.001^∗∗^	0.937 (0.694, 1.264)	0.669
Heparin_group	3.209	<0.001^∗∗^	1.033 (0.946, 1.128)	0.472
Hydroxychloroquine	0.933	<0.001^∗∗^	0.850 (0.787, 0.918)	<0.001^∗^

Positive mean differences indicate further risk for exposure positive (for age, age ≥ 50 was regarded). The regression model has been adjusted with age interactions. NA: not applicable. ^∗^Significant at 0.05 (Wald test). ^∗∗^Significant at 0.001 (*t*-test, bonferroni correction).

## Data Availability

The data cannot be taken out offline from the datacenter due to security and ethical reasons. SAYA and AK are available to perform further requested analyses (ahmadi.say@iums.ac.ir, kabir.a@iums.ac.ir).

## References

[1] Zhang S., Li L., Shen A., Chen Y., Qi Z. (2020). Rational use of tocilizumab in the treatment of novel coronavirus pneumonia. *Clinical Drug Investigation*.

[2] Shi Y., Wang Y., Shao C. (2020). COVID-19 infection: the perspectives on immune responses. *Cell Death & Differentiation*.

[3] Kosmaczewska A., Frydecka I. (2020). Dysregulation of the immune system as a driver of the critical course of the novel coronavirus disease 2019. *Polish Archives of Internal Medicine*.

[4] Tang L., Yin Z., Hu Y., Mei H. (2020). Controlling cytokine storm is vital in COVID-19. *Frontiers in Immunology*.

[5] Wu R., Wang L., Kuo H.-C. D. (2020). An update on current therapeutic drugs treating COVID-19. *Current Pharmacology Reports*.

[6] Smith G. (2018). Step away from stepwise. *Journal of Big Data*.

[7] Hariyanto T. I., Putri C., Situmeang R. F. V., Kurniawan A. (2020). Dementia is a predictor for mortality outcome from coronavirus disease 2019 (COVID-19) infection. *European Archives of Psychiatry and Clinical Neuroscience*.

[8] Simmons B., Wentzel H., Mobarak S. (2021). Sofosbuvir/daclatasvir regimens for the treatment of COVID-19: an individual patient data meta-analysis. *Journal of Antimicrobial Chemotherapy*.

[9] Brandariz-Nuñez D., Correas-Sanahuja M., Guarc E., Picón R., García B., Gil R. (2020). Interacciones medicamentosas potenciales en pacientes COVID 19 en tratamiento con lopinavir/ritonavir. *Medicina Clínica (English Edition)*.

[10] Alhumaid S., al Mutair A., al Alawi Z., Alhmeed N., Zaidi A. R. Z., Tobaiqy M. (2020). Efficacy and safety of lopinavir/ritonavir for treatment of COVID-19: a systematic review and meta-analysis. *Tropical Medicine and Infectious Disease*.

[11] Bhattacharyya A., Kumar S., Sarma P. (2020). Safety and efficacy of lopinavir/ritonavir combination in COVID-19: a systematic review, meta-analysis, and meta-regression analysis. *Indian Journal of Pharmacology*.

[12] Shrestha D. B., Budhathoki P., Khadka S., Shah P. B., Pokharel N., Rashmi P. (2020). Favipiravir versus other antiviral or standard of care for COVID-19 treatment: a rapid systematic review and meta-analysis. *Virology Journal*.

[13] Wu C., Lin Z., Ye Y. Oseltamivir, lopinavir/ritonavir and reduning may improve survival of COVID-19 patients with high-risk.

[14] Gharebaghi R., Heidary F., Moradi M., Parvizi M. (2020). Metronidazole; a potential novel addition to the COVID-19 treatment regimen. *Archives of Academic Emergency Medicine*.

[15] Kumar R., Kumar V., Lee K. W. (2021). A computational drug repurposing approach in identifying the cephalosporin antibiotic and anti-hepatitis C drug derivatives for COVID-19 treatment. *Computers in Biology and Medicine*.

[16] Fiolet T., Guihur A., Rebeaud M., Mulot M., Peiffer-Smadja N., Mahamat-Saleh Y. (2021). Effect of hydroxychloroquine with or without azithromycin on the mortality of coronavirus disease 2019 (COVID-19) patients: a systematic review and meta- analysis. *Clinical Microbiology and Infection*.

[17] Hariyanto T. I., Japar K. V., Kwenandar F. (2021). Inflammatory and hematologic markers as predictors of severe outcomes in COVID-19 infection: a systematic review and meta-analysis. *The American Journal of Emergency Medicine*.

[18] Lugito N. P. H. (2021). Is procalcitonin a part of human immunological response to SARS-CoV-2 infection or "just" a marker of bacterial coinfection?. *Current Research in Translational Medicine*.

[19] WHO Rapid Evidence Appraisal for COVID-19 Therapies (REACT) Working Group, Sterne J. A. C., Murthy S. (2020). Association between administration of systemic corticosteroids and mortality among critically ill patients with COVID-19: a meta-analysis. *Journal of the American Medical Association*.

[20] RECOVERY Collaborative Group, Horby P., Lim W. S. (2021). Dexamethasone in hospitalized patients with Covid-19. *New England Journal of Medicine*.

[21] Russell B., Moss C., George G. (2020). Associations between immune-suppressive and stimulating drugs and novel COVID-19—a systematic review of current evidence. *ecancermedicalscience*.

[22] Edalatifard M., Akhtari M., Salehi M. (2020). Intravenous methylprednisolone pulse as a treatment for hospitalised severe COVID-19 patients: results from a randomised controlled clinical trial. *European Respiratory Journal*.

[23] Liu J., Zheng X., Huang Y., Shan H., Huang J. (2020). Successful use of methylprednisolone for treating severe COVID-19. *Journal of Allergy and Clinical Immunology*.

[24] Wang M., Wu T., Zuo Z. (2021). Evaluation of current medical approaches for COVID-19: a systematic review and meta-analysis. *Supportive & Palliative Care*.

[25] Bae M., Kim H. (2020). The role of vitamin C, vitamin D, and selenium in immune system against COVID-19. *Molecules*.

[26] Baladia E., Pizarro A. B., Rada G. (2020). Vitamin C for the treatment of COVID-19: A living systematic review. https://www.medrxiv.org/content/10.1101/2020.04.28.20083360v2/.

[27] Singh A. K., Singh A., Singh R., Misra A. (2020). Hydroxychloroquine in patients with COVID-19: a systematic review and meta- analysis. *Diabetes & Metabolic Syndrome: Clinical Research & Reviews*.

[28] Pathak S. K., Salunke A. A., Thivari P. (2020). No benefit of hydroxychloroquine in COVID-19: results of systematic review and meta-analysis of randomized controlled trials. *Diabetes & Metabolic Syndrome: Clinical Research & Reviews*.

[29] Group RC (2020). Effect of hydroxychloroquine in hospitalized patients with Covid-19. *New England Journal of Medicine*.

[30] Lobastov K., Schastlivtsev I., Porembskaya O., Dzenina O., Bargandzhiya A., Tsaplin S. (2020). COVID-19-associated coagulopathy: review of current recommendations for diagnosis, treatment and prevention. *Нospital-Replacing Technologies: Ambulatory surgery*.

